# Testing of the siderophore deferoxamine amended in hydrogels for the cleaning of iron corrosion

**DOI:** 10.1140/epjp/s13360-023-04159-y

**Published:** 2023-06-27

**Authors:** Luana Cuvillier, Arianna Passaretti, Elodie Guilminot, Edith Joseph

**Affiliations:** 1 Laboratory of Technologies for Heritage Materials, University of Neuchâtel, Bellevaux 51, 2000 Neuchâtel, Switzerland; 2 Haute Ecole Arc Conservation Restauration, University of Applied Sciences and Arts Western Switzerland HES-SO, Espace de l’Europe 11, 2000 Neuchâtel, Switzerland; 3 Arc’Antique Conservation and Research Laboratory, 26 Rue de la Haute Forêt, 44300 Nantes, France

## Abstract

Bioderived alternatives to commonly used complexing agents for the cleaning of iron artworks are sought for their natural origin and better biodegradability. Indeed, complexing agents currently used for the removal of undesired corrosion products from iron artworks can be difficult to control and their environmental impact is often overlooked. This paper studies the use of siderophores, focusing on the ability of one of them, deferoxamine, to be employed as an active agent loaded in polysaccharides hydrogels, on corrosion phases. Preliminary tests were conducted on artificially aged steel samples and further studies were performed on naturally corroded steel to assess the most performing application parameters. Long-term behavior of cleaned surface was assessed. Cleaning outcomes were compared with those obtainable with disodium ethylenediaminetetraacetic acid using optical microscopy, colorimetry and atomic absorption spectroscopy as well as Infrared and Raman micro-spectroscopies. Among the different gelling agents evaluated, agar applied when hot and gellan gum prepared at room temperature were the most effective gel formulations and agar left few residues over the treated surfaces. The protocol was then tested on altered steel artifacts belonging to heritage institutions in France. Encouraging outcomes in the removal of iron corrosion phases with green approaches are here presented.

**Graphical abstract:**

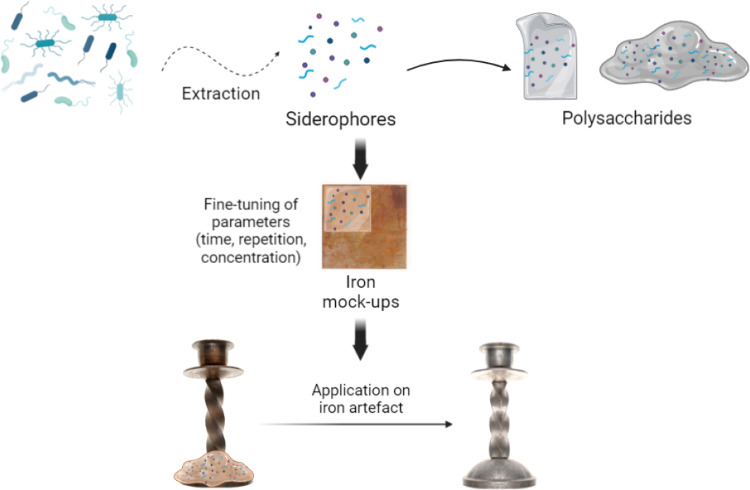

## Introduction

A global impulse drives researchers to investigate more sustainable alternatives, including using naturally synthesized compounds rather than petrochemically synthesized ones. The corrosion phenomenon makes no exception especially in the field of cultural heritage [1–3]. Corrosion is a natural phenomenon affecting metals, which implies great safety and economical stakes worldwide. Iron artworks are also very much concerned by the issue. Indeed, for archeological or historical objects stored in museums, the removal of active or inaesthetic corrosion is needed to arrest ongoing damage processes or to facilitate the appreciation, readability, or use of the object itself. In this paper, the work is focused on the preservation of iron heritage where removal of all or part of the corrosion layer is sought. New methods have emerged for the care of such artifacts that imply the use of complexing agents in gel formulations. Indeed, the use of gels has become relevant because of their ease of application, allowing a controlled cleaning and a lowered amount of active substances used [4, 5]. Numbers of polysaccharide-based hydrogels are originated from natural compounds (i.e., metabolites from bacteria or fungi, cell walls from mushrooms or seaweeds). Among several alternatives of complexing agents that can be used Ethylenediaminetetraacetic Acid Disodium Salt (EDTA) is one of the preferred options for conservators. Unfortunately, although the quantities used in heritage conservation are much smaller than in other industrial applications, EDTA is not biodegradable and its environmental impact is a worldwide concern [6]. In addition, despite its easy availability and low cost, EDTA is classified as an irritant and it must be used with appropriate caution [7]. As alternative, siderophores are natural iron-specific complexing agents which could be a reliable option. They are secondary metabolites used to fetch iron in iron-deficient environments to ensure the right development of the microorganism [8]. Siderophore production under iron-deficient conditions has been detected in many genera of microbes [8].

Siderophores can be classified into three main types depending on their iron-binding functional group: catecholates, hydroxamates, and carboxylates. In addition, some of them display more than one of the moieties stated before; these are considered as mixed types [9]. Siderophores are secreted out to acquire iron in an environment where it is lacking. Briefly, these metabolites can uptake iron forming a ferri-siderophore complex that is recognized and translocated inside cells. It is believed that the reason the siderophore will release the iron from the complex is a reduction from Fe^3+^ to Fe^2+^ inside the cell, as the affinity of siderophores for ferrous iron is lower than for ferric iron. Fe^3+^ is a stronger Lewis acid than Fe^2+^, therefore the electron-pair donor groups present in the siderophores bind ferric rather than ferrous iron [10]. The affinity can be evaluated thanks to the stability constant logβ of each siderophore with metallic ions, which is defined using the ratio between formed complexes and free ions. The higher logβ, the more stable the complex. In opposition to other complexing agents such as EDTA, siderophore show a specific affinity for iron, albeit they can complex other ions but to a lower extent [11]. Table 1 shows different formation constants of general siderophores with common elements in metallic cultural heritage [10–13]. The variety of siderophores and siderophore-types in nature shows an interesting potential ability to fine-tune the treatment according to the need and targeted product. For instance, a siderophore having rather high affinities with other ions might not be suitable for the cleaning of painted metals, as it could impair the paint layer by chelating pigments.

**Table 1:** Characteristics of siderophores produced by bacterial strains *Streptomyces pilosus*, *Pseudomonas fluorescens*, *Escherichia coli* in comparison with Na_2_EDTA

Strain	*Streptomyces pilosus*	*Pseudomonas fluorescens*	*Escherichia* coli	EDTA
Main siderophore produced	Deferoxamin	Pyoverdin	Enterobactin	n/a
Siderophore type	Hydroxamate	Mixed	Catecholate	n/a
Logβ Fe^3+^	30.6	30.8	49	25.1
Logβ Fe^2+^	10.2	9.78	23.9	14.3
Logβ Cu^2+^	14.1	14.9		18.8
Logβ Zn^2+^	10.1	11.8		16.5

In this paper, the siderophore deferoxamine, from the bacterial strain *S.pilosus* was selected. Even though enterobactin, the main siderophore of *E. coli*, is known to have the highest iron binding affinity, or stability constant (logβ = 49), deferoxamine (logβ = 30.6) is also well described in literature, easily available, and has been used in heritage studies for the removal of iron [11, 14, 15]. It is an hydroxamate-type siderophore, hexadentate ligand, with a complexing DFO-Fe ratio of 1:1 and a rather neutral pH [11]. In addition, the DFO-Fe complex displays a vivid orange color [16], which allows an easier monitoring of the complex formation and thus of the treatment.

Evaluation of gelled siderophore solutions for cleaning iron corrosion layer was performed on both artificially and naturally aged samples. Several parameters were considered (e.g., time of application, concentration of active agents and long-term behavior of treated sample over time). Due to the numerous existing and used gelling agents, assessment of siderophore efficiency was performed using different hydrogels matrixes. Three polysaccharides, widely used in conservation (i.e., xanthan, gellan gum and agar), were compared in terms of their impact on the cleaning efficiency. Finally, a test on two case studies allowed to evaluate the possible implementation in real praxis.

## Materials and methods

### Heating resistance of siderophores

The heat resistance of selected siderophores was checked to ascertain the feasibility to prepare a gel containing those, making sure they do not lose their complexing abilities after reaching high temperature (> 100 °C). The heating resistance of a siderophore, deferoxamine (DFO), (Desferal®, Novartis) was evaluated using UV–visible spectrometry by examining the iron-siderophore complex formation before and after boiling of a solution prepared as a mix of Deferoxamine (100 µL) and a ferric nitrate solution (Fe(NO_3_)_3_·9H_2_O) (100 µL) at the same molar concentration, 10^−2^ M, and diluted 4 times if necessary.

### Treatment evaluation

For reproducibility purpose, each experiment was performed on triplicates, exception made where mentioned differently.

#### Samples

Two types of samples were employed. Steel samples (30 × 30 × 1 mm) were obtained from Tartaix and artificially corroded adapting ASTM G48-11 procedure by immersing the samples into 0.4 M FeCl_3_ and then 9.79 M H_2_O_2_ to accelerate corrosion formation. Also naturally corroded mild steel samples (20 × 30 × 2 mm) were obtained from a conservation workshop (Arc’Antique, Nantes, France), where over a period of years they underwent corrosion due to uncontrolled indoor conditions. The corrosion layer, characterized beforehand by Raman spectroscopy, was composed of both goethite and lepidocrocite, common compounds that develop during indoor iron corrosion [17, 18].

#### Preliminary cleaning evaluation

The use of pure compounds is favored when applied to cultural heritage conservation to avoid any possible interference with culture media or other metabolites produced by the microorganisms [10].

Hence, it was decided to use readily purified Deferoxamine, available under its commercial form, Desferal® in this study.

Preliminary test on artificially corroded iron samples were performed to assess the uptake of insoluble iron phases. A 3% w/v agar gel amended with a 3×10^−2^ M DFO water solution was applied for 20 min on samples (triplicates). This gel was also applied on a non-corroded sample to evaluate the aggressiveness of DFO on base iron. Similar process was performed using an EDTA-water solution, at the same concentration (PanReac Applichem). A sample where plain 3% w/w agar gel (not amended with siderophores) was applied was used as control. Samples were characterized by means of optical microscopy, Eddy current measurements, colorimetry and Fourier Transformed Infrared (FTIR) spectroscopy before and after gel applications.

#### Study of application parameters

Application parameters and kinetics were studied on naturally corroded iron samples in order to have a homogeneously spread corrosion and therefore comparable results.

A 3% w/v agar gel with DFO water solutions at concentrations of 0, 3×10^−4^, 3×10^−3^, 3×10^−2^ and 6×10^−2^ M was applied to naturally corroded iron samples for 10 min. The application was repeated six times for a total application time of 60 min. For comparison, a 3% w/v agar gel amended with a 6×10^−2^ M water solution of Na_2_EDTA was tested using the same reiterative protocol. Samples were characterized by means of colorimetry and optical microscopy before and after gel application. Atomic Absorption Spectroscopy (AAS) of the gels was performed to ascertain the iron contained in the gel after application.

For the cleaning kinetics and eventual influence of the gel matrix used, iron chelation was assessed after immersion of the naturally corroded iron samples for 10 and 30 min, 1 h, 5 h and 24 h in a 6×10^−2^ M solution of DFO or after application of a 3% w/v agar DFO-gel with the same parameters (concentration and application duration). The experiments were carried out at room temperature, approximately 20 °C. Immersion solution and gels were analyzed using AAS comparing their iron content before and after test.

### Behavior of cleaned surfaces over time

Cleaned naturally corroded samples (triplicates) were stored for a year under uncontrolled indoor conditions in Nantes, France. Temperature (T) and relative humidity (RH) variation in the storage place over the year are, respectively, 17 °C < T < 21 °C and 45% < HR < 60%. Colorimetric and visual evaluations were performed after one year to ascertain the stability of the treatment.

### Selection of best-performing gel formulation

Three naturally derived hydrogels amended with a 6×10^−2^ M solution of DFO were assessed on the naturally corroded iron samples from the workshop. Several application methods are evaluated as detailed in Table 2. Each gel formulation was applied for 10 min and removed using a cotton swab dipped in 70% v/v ethanol (Table 3).

**Table 2:** Gel formulations amended with 6·10^−2^ M solution of DFO and evaluated for the cleaning of naturally corroded iron samples

Gelling agent	Xanthan gum	Agar–agar		Gellan gum	
Brand, supplier	Vanzan©, CTS	AgarArt©, CTS		Phytagel™, Sigma Aldrich	
Source	*Xanthomonas campestris*	*Gelidium* and *Gracilaria* red seaweeds		*Sphingomonas elodea*	
Preparation	5% w/v in H_2_O and stirring	3% w/v in H_2_O, heated to 90 °C twice		3% w/v in H_2_O, stirring	3% w/v in H_2_O, heated to 90 °C
Application	With a spatula at room temperature	Dripped when still hot	Cooled preformed rigid foil	With a spatula at room temperature	Dripped when still hot
Texture	Viscous	Rigid, peelable	Rigid	Viscous	Rigid, peelable

**Table 3:** Iron-based objects selected as case studies

	Objects	
		
Description	Candelholder Historical piece Thin corrosion layer	Helmet Archeological object Sediments and corrosion compounds displayed in its stratigraphy
Provenance	Diocese of Nantes (France)	Dobrée Museum (Nantes, France)
Application	2 × 15’	3 × 20’

### Case studies

Two iron-based objects were selected to evaluate the application of siderophore-based cleaning gel formulations. A 3% w/v agar gel amended with 6×10^−2^ M of deferoxamine, or 6×10^−2^ M of EDTA was applied on different areas of the four objects as well as a plain 3% w/v agar gel as control. Either visual observations and or Raman measurements were achieved to evaluate the cleaning efficiency.

### Analytical techniques

#### Colorimetry

A Minolta CM-508D spectrophotometer was used for colorimetry measurements. The setup was as follows: specular component excluded (SCE), illuminant D65, d/8° geometry, 10° observer, window size 8 mm, and CIELab color space. Measurements were performed as triplicates per chosen area.

ΔE* was calculated using the standard color variation formula in CIELab where L*_1_, a*_1_ and b*1 are the coordinates in the colorimetric space of the first measurement, and L*_2_, a*_2_, b*_2_ those of the second measurement. Triplicated measurements.

$$\Delta E^{*} = \sqrt {(L_{2}^{*} - L_{1}^{*} )^{2} + (a_{2}^{*} - a_{1}^{*} )^{2} + (b_{2}^{*} - b_{1}^{*} )^{2} }$$

ΔE*, the total color difference index provides an overall estimation of the color difference but is not representative of the qualitative difference (L*, a*, b*) of the color changes. For that reason, graphs showing the three color coordinates were preferred. ΔL, Δa and Δb were calculated using the following formula: Δ*α* = *α*_after_-*α*_before,_ where α_after_ is L*, a* or b* after treatment and α_before_ is L*, a* or b* before treatment.

#### Optical microscopy

Optical microscopy images were acquired using a Leica DMS1000 Digital microscope system with Leica Application Suite software.

#### Atomic absorption spectroscopy

A Thermo Fisher iCE 3300 double-beam atomic absorption spectrometer equipped with an acetylene-air flame was used to determine the amount of iron ions present in gel formulations or DFO solutions after treatment. The gels were first dissolved in 10 mL of 70% v/v HNO_3_ and diluted with deionized water to a final volume of 100 mL. The solutions were not filtered, in order to allow a determination of the total amount of iron present including both the ones complexed but also the ones that were stripped from the surface by the mechanical peeling action of the gel. Measurements were performed as triplicates. Obtained results were normalized by the weight of each gel to have comparable results.

#### Ultra–violet-visible spectroscopy

UV–visible data were acquired with a VICTOR Nivo Multimode Microplate Reader from PerkinElmer in the visible range 400–850 nm.

#### Fourier transformed infrared spectroscopy

A Thermo Fisher Nicolet iN10MX was used in reflectance mode. FTIR Spectra were recorded with the following parameters: 650–4000 cm^−1^ spectral range, 4 cm^−1^ resolution, 16 scans. Post-processing of the spectra was achieved with OMNIC software. Baseline correction and atmospheric correction were performed to remove residual signatures of atmospheric CO_2_ and water in the spectra.

#### Eddy current measurements

A Surfix Pro S coating thickness gauge equipped with an FN 1.5/90° right angle probe was employed as follows: 5 mm Ø measurement sensor, 1 ± 0.1 µm precision, was used to get the Eddy current value, indicating the corrosion layer thickness. Measurements were performed as triplicates.

#### Raman spectroscopy

A Jobin Yvon Horiba T640000 Raman microspectrometer equipped with 50 × magnification objective was employed to examine the helmet using a 633 nm laser with a laser power of 0.1 mW. Spectra were recorded in the spectral range of 100–1500 cm^−1^ with four accumulations of 30 s each. The data were post-processed using OMNIC software.

## Results and discussion

### Heat-resistance of siderophores

**Fig. 1:**
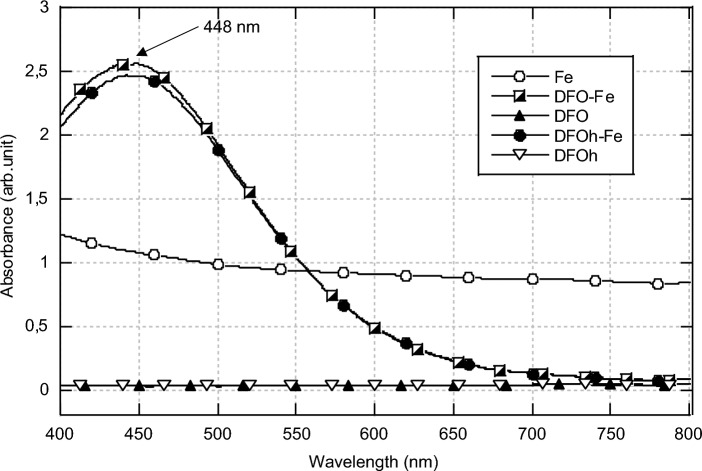
Visible spectra (400–850 nm) of solubilized iron(III) (Fe), deferoxamine (DFO), deferoxamine after heating (DFOh), iron-deferoxamine complex (DFO-Fe) and iron-deferoxamine complex after heating of deferoxamine (DFOh-Fe)

The compatibility of deferoxamine with gel formulations that would require a heating step is demonstrated. The iron-deferoxamine complex is known to absorb at 448 nm [19]. From the obtained absorbance spectra (Fig. 1, spectrum DFOh-Fe), it is clear that the siderophore deferoxamine still possesses chelating properties after reaching temperatures over 100 °C, confirming literature data [20]. Indeed, the characteristic absorbance peak of the DFO-Fe complex at 448 nm is still present.

### Treatment evaluation

#### Preliminary cleaning evaluation

The application of a 3% w/v agar gel amended with DFO on non-corroded steel showed no visual modification. In addition, the colorimetric values of the sample prior to the application of the gel were left unchanged, suggesting the absence of interaction of the siderophores with the base iron (Table 4). Indeed, siderophores rather target ferric iron [10].

**Table 4:** Optical microscopy observation and colorimetric values of bare iron samples before and after 3% w/v Agar-DFO gel application

	Before cleaning			After cleaning		
Colorimetric values	L*	a*	b*	L*	a*	b*
	*65.67* ± *0.28*	0.74 ± 0.43	3.36 ± 1.36	*66.71* ± *0.85*	0.83 ± 0.12	3.88 ± 0.64

Scale bar indicates 2 mm

**Fig. 2:**
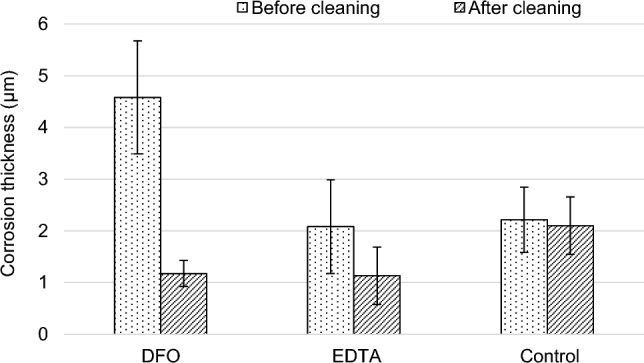
Corrosion layer thickness before (dotted) and after (stripped) application of a 3% w/v Agar gel amended with 6×10^−2^ M solution of DFO or 6×10^−2^ M solution of EDTA. 3% w/v plain gel without complexing agent serves as control

Gels application on artificially aged steel samples allowed to remove the corrosion layer, as shown by digital microscope observations (Table 5). A clear decrease in the corrosion layer thickness was confirmed by Eddy current measurements (Fig. 2). The inhomogeneity of the artificially created corrosion layer is to be noted, whether from visual or thickness point of view, thus leading to the use of naturally corroded samples in the following sections of the study.

**Table 5:** Artificially corroded steel samples before and after 3% w/v Agar-DFO or Agar-EDTA gel application

	Before cleaning	After cleaning
DFO		
EDTA		
Control		

Scale bar indicates 2 mm

**Fig. 3:**
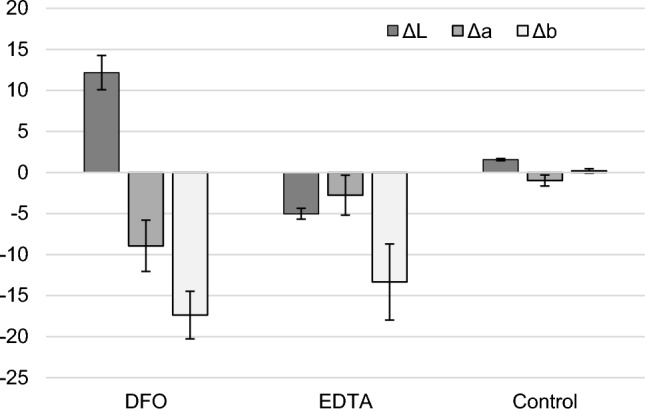
Variations of colorimetric coordinates of the artificially corroded iron samples before and after application of a 3% w/v Agar gel amended with 6×10^−2^ M solution of DFO(DFO) or 6×10^−2^ M solution of EDTA. 3% w/v plain gel without complexing agent serves as control

The aim of a cleaning intervention is to retrieve, as much as possible, the appearance of the metal before corrosion. Regarding the results obtained through the three color coordinates, it appears that lightness L* increased in samples cleaned with deferoxamine-based gel formulation to reach a value of L* = 58.23 ± 2.86 after treatment (Fig. 3). Samples treated with EDTA-amended agar gel on the contrary turned darker as shown with a negative ΔL* value, reaching a value of L* = 48.41 ± 2.25 further from the bare steel (L* = 65.67 ± 0.28). This is possibly due to its acidic pH of 4.2 at 6·10^–2^ M (vs a pH of 6.8 for DFO), which can alter metallic iron [21]. In both cases, the change of color is good as it bears witness of the removal of rust. The final appearance is up to the conservator and restorer according to the look desired. a* and b* coordinates were both shifted toward lower values, indicating that the cleaned surfaces turned less yellow and less red, as expected with the removal of iron corrosion products. Color variations on the control corroded samples where a plain gel was applied are not observed (Δb) or very low (ΔL and Δa). The low variation for the control samples could be explained some iron being stripped off when peeling off the agar gel from the surface.

**Fig. 4:**
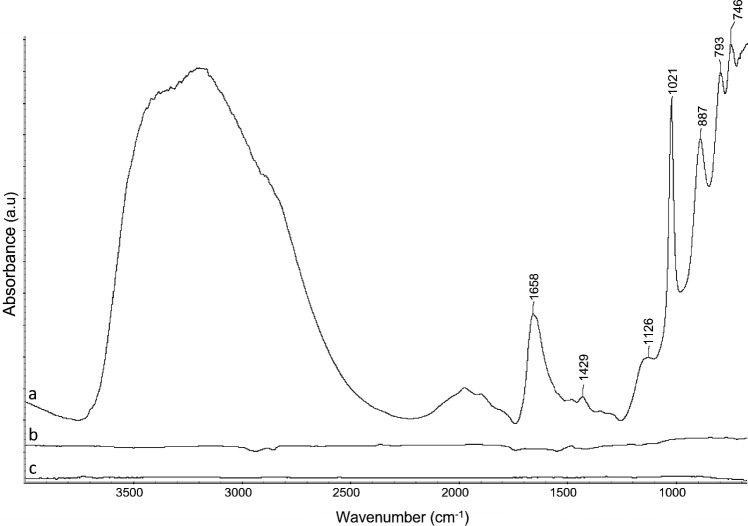
FTIR spectra of artificially aged samples before treatment (**a**), after treatment (**b**) and bare iron sample (**c**)

In addition, FTIR spectra of the artificially corroded iron samples after cleaning did not display the characteristic bands of iron corrosion products identified before treatment. Similar spectrum as bare iron samples were obtained (Fig. 4). Band attribution can be read in Table 6.

**Table 6:** Assignment of the vibrational bands observed in the FTIR spectra of artificially corroded iron sample (Fig. 4a)

Wavelength (cm^−1^)	Assignment	References
746	Lepidocrocite	[22]
793	Goethite	[22]
887	Goethite	[22]
1021	Lepidocrocite	[22]
1126	Lepidocrocite	[22]
1429	Iron oxy-hydroxides	[23]
1658	Iron oxy-hydroxides	[23]
3000–3500	Iron oxy-hydroxides	[23]

#### Study of application parameters

**Fig. 5:**
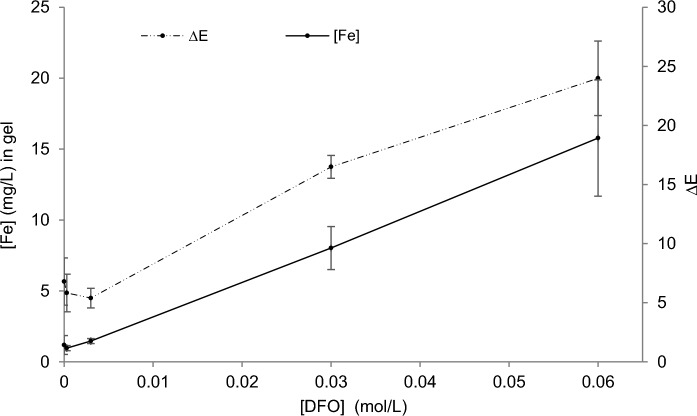
Iron content of 3% W/v agar gels amended with 0, 3×10^−4^, 3×10^−3^, 3×10^−2^ or 6×10^−2^ M of DFO solution, after a 10 min application. Color difference measured before and after application of gel formulations

After gel application of DFO at different concentrations on naturally corroded steel, colorimetric results on the samples show an important hue change at concentration starting from 3×10^−2^ M (Fig. 5). Results were confirmed by AAS analyses of the gels after their use on these sample, where iron concentration increases rapidly at a concentration of 3×10^−2^ M and then proportionally with the DFO concentration. Thus, a 6×10^−2^ M concentration was selected for the further experiments described below, as it allows a greater performance.

**Fig. 6:**
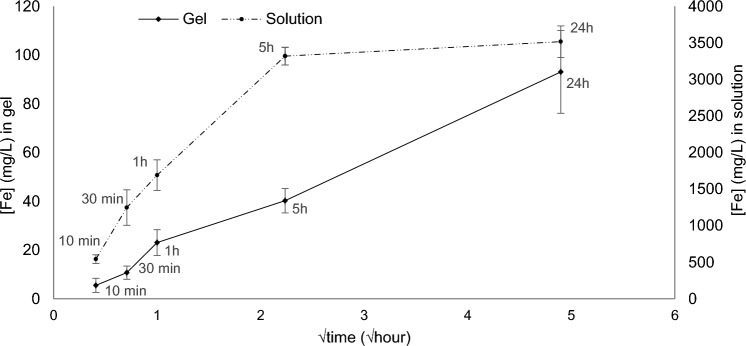
Iron uptake by a 3% w/v agar gel amended with 6×10^−2^ M solution of DFO in contact with naturally corroded iron (plain line) and a 6×10^−2^ M DFO water solution after immersion of naturally corroded iron samples (dotted line) for 10 min, 30 min, 1 h, 5 h and 24 h at 20 °C

Deferoxamine theoretically binds with iron at a 1:1 molar ratio, meaning that a 6×10^−2^ M DFO solution can uptake up to 3.3 g/L of iron, as confirmed with the plateau reached for iron concentration in Fig. 6.

Figure 6 shows a threshold in the iron uptake reaction after 5-h contact of naturally corroded iron sample with a 6×10^−2^ M DFO water solution that is not present when using a gel matrix. This can be explained by the diffusion of DFO molecules within the agar gel, as described by the Higuchi model which states that release from a matrix is a square root of time-dependent process and is based on Fick’s law of diffusion [24]. It is indeed the case for the Agar-DFO gel which follows a linear regression when plotted versus the square root of time.

**Fig. 7:**
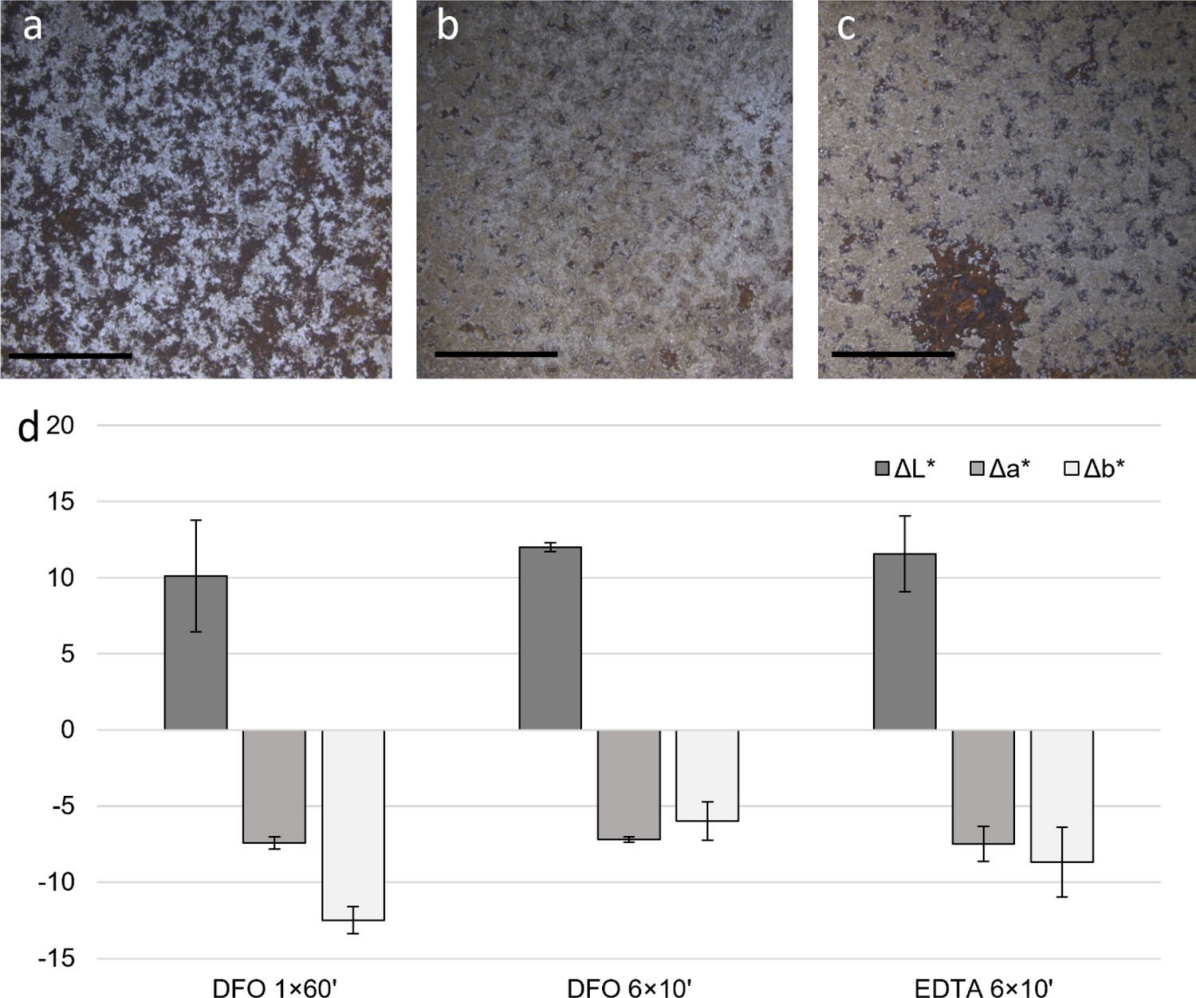
Digital microscope observations of naturally corroded iron samples after the application of a 3% w/v Agar gel amended with 6×10^−2^ M solution of DFO for 60 min **a**, for 6 × 10 min **b** and of 3% w/v Agar gel amended with 6×10^−2^ M solution of EDTA for 6 × 10 min **c**, and corresponding color differences ΔL*, Δa* and Δb* calculated for the 3 different gels application **d**. Scale bar indicates 5 mm

**Fig. 8:**
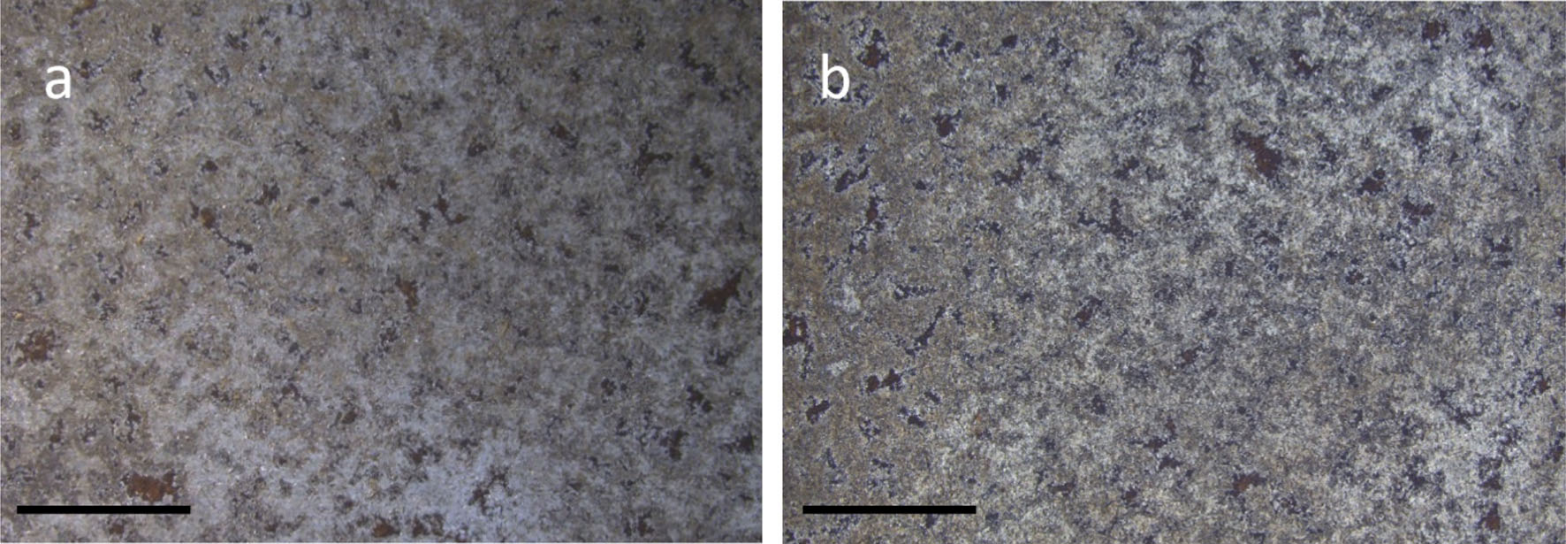
Naturally aged iron sample immediately after Agar-DFO treatment **a** and one year after treatment **b**. Scale bar indicates 5 mm

In addition, the iron uptake rate seems greater in the first period of contact with the gel (Fig. 8). This suggests that for a better cleaning performance of the gel formulation, reiteration of short time applications is preferred. It is worth mentioning that end-users also prefer this reiteration to have a greater control of their intervention. On Fig. 7, the differences according to the number of reiterations of the gel application on naturally corroded iron sample can be observed, where six gel applications of 10 min performing better than a unique application of 60 min. The results with EDTA show equivalent results (Fig. 7c). These results are confirmed by colorimetry values; indeed lightness is increased in a more efficient way when the gel is applied 6 times for 10 min rather than one time 60 min. DFO and EDTA also show similar performance when regarding the color difference before and after application, confirming that successive smaller applications provide a more effective cleaning than a long one (Fig. 7d).

### Stability of the treated pieces over time

Average color difference ΔE* of the naturally aged iron samples after treatment and after one year stored in indoor and uncontrolled conditions was of 3.33 (± 1.69) on areas treated with DFO-amended agar gel and 4.25 (± 1.73) for EDTA-amended agar gel. ΔE values obtained for both DFO and EDTA are inferior to 5, such color differences are not perceptible to the human eye [25]. In addition, digital microscope observations showed a similar surface appearance after the 1-year storage (Fig. 8).

Although the removal protocol should ensure the absence of residues from the treating gel and solution, Recent studies present deferoxamine as a reliable corrosion inhibitor, thanks to its hydroxamate group, allowing to underline its potentialities further [9].

### Selection of best-performing gel formulation

**Fig. 9:**
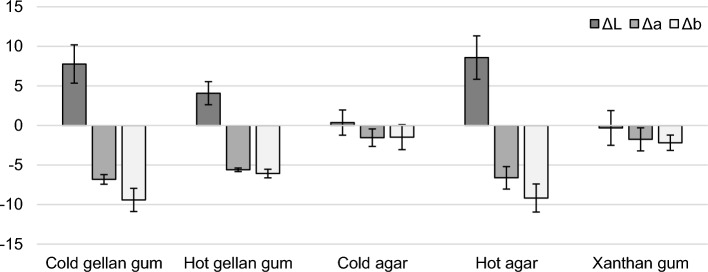
Color differences ΔL*, Δa* and Δb* measured on the naturally aged iron samples before and after cleaning using different gel matrixes (Cold prepared and applied gellan gum, hot applied gellan, cold and hot applied agar and xanthan gum) loaded with 6×10^−2^ M DFO solution

On Fig. 9, L*a*b* coordinates of naturally aged iron samples before and after cleaning showed that best outcomes in terms of corrosion removal were obtained with 3% w/v agar gel applied hot and a gellan gel prepared and applied cold (at room temperature). Hot-applied gellan gel formulation achieved mild cleaning. Performances of DFO-xanthan gel as well as the rigid DFO-agar gel applied cold were the poorest. After cleaning with DFO-loaded hot applied agar gel, room temperature gellan gel and to a lesser extent hot gellan gel, the surface color was brighter (higher values of L*) and the hue turned to blue-green (higher values of a* and b*). This can be attributed to the removal of the red–orange iron corrosion products. By contrast, low surface color changes were seen on the samples treated with DFO-amended gels based on xanthan gum or cold agar. After xanthan gum application, a thin layer of gel remained that could not be removed by cotton swab rinsing. For the applied cold rigid DFO-amended agar gel, no clear visual modifications were observed, probably due to the failure of the cold and rigid agar matrix to adhere to the surface and achieve a close contact with the corroded iron surface [5]. Despite its great cleaning performances and potential, DFO-amended gellan gum gel at room temperature needs extra care to be observed at the removal because its texture implies the need for a careful rinsing after removal, especially when applied to carvings in the case of potential applications on objects.

### Application on case studies

**Fig. 10:**
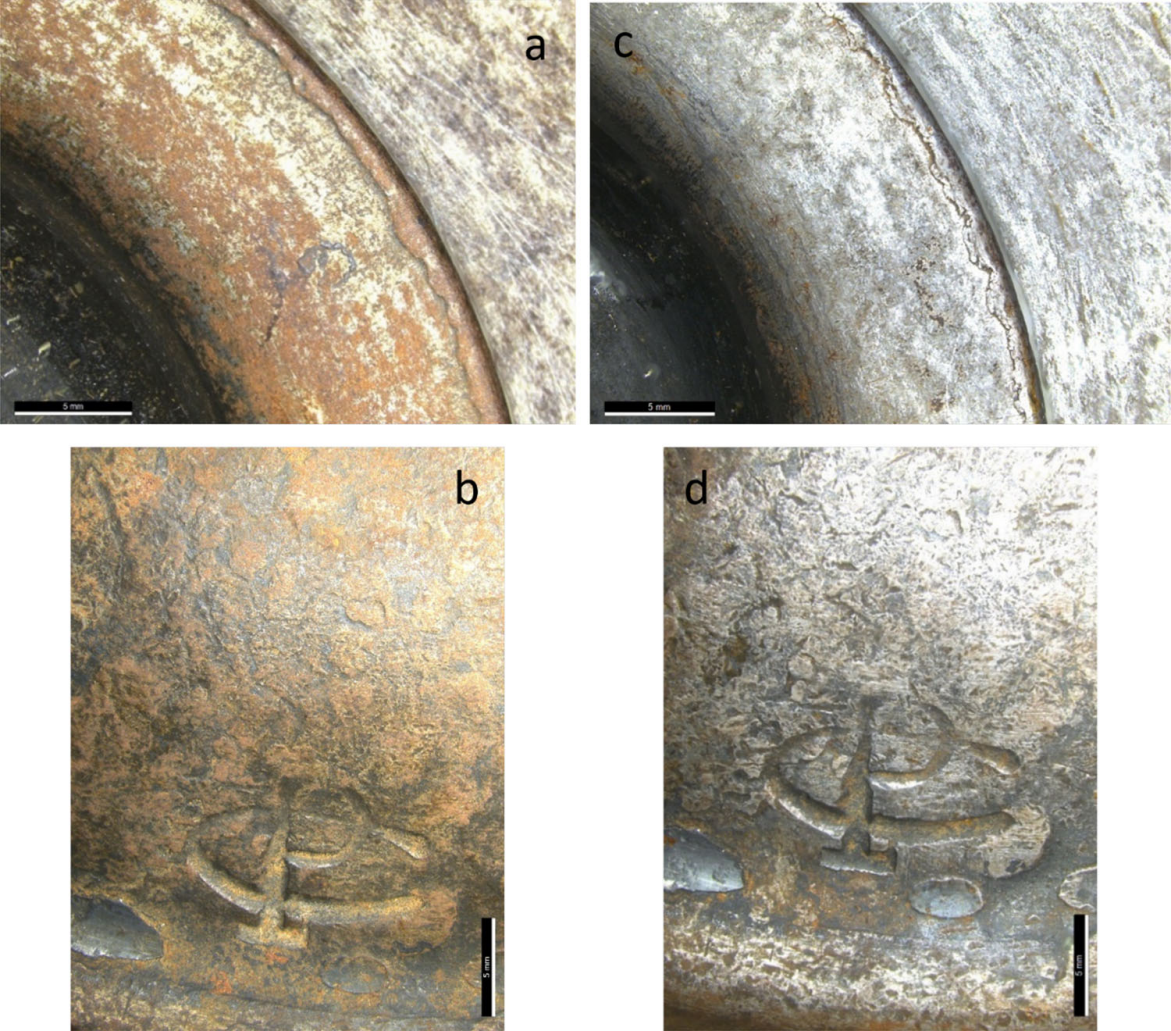
Digital microscope images of details from the candleholder before **a**, **b** and after **c**, **d** cleaning. Scale bar indicates 5 mm

Visual observations of the candleholder indicated an efficient cleaning with DFO-amended 3% w/v agar gel as it performed better than other tested gelling agents. The removal of the iron corrosion products revealed the underlying metal and the objects’ details (Fig. 10).

On the helmet, the presence of sediments and thus calcium-based compounds resulted in the DFO-amended 3% w/v agar gel being less effective. Unlike EDTA, that can bind iron and calcium (logβ_Fe3+-EDTA_ = 25 and logβ_Ca2+-EDTA_ = 10.9), DFO is specific for iron and its affinity for calcium ions is poor (logβ_Fe3+-DFO_ = 30.6 and logβ_Ca2+−DFO_ ≤ 3.03) [11, 26, 27].

**Fig. 11:**
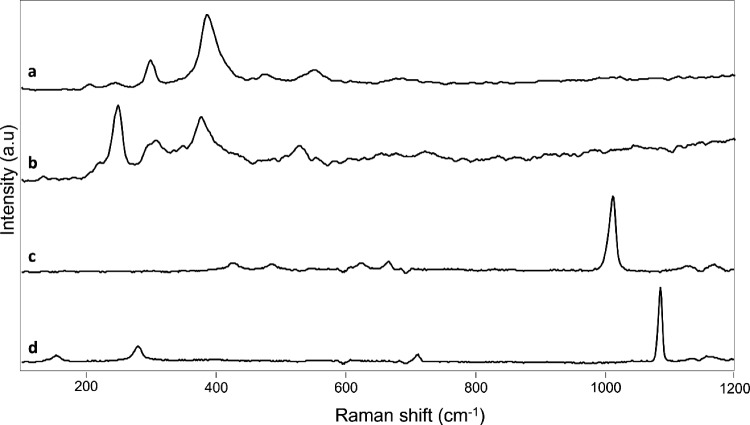
Raman spectra of goethite **a**, lepidocrocite **b**, anhydrite **c** and calcite **d** obtained on the helmet

Raman analyses performed after cleaning confirmed and identified the presence of calcium-based compounds (e.g., CaSO_4_, CaCO_3_) along with iron oxy-hydroxides on the areas treated with DFO-amended 3% w/v agar gel but not with EDTA-amended 3% w/v agar gel (Fig. 11) [28, 29]. Although less sustainable, EDTA offers the benefit of tackling sediments as well as corrosion products in one step.

DFO’s specific affinity for iron supports the use of this compound as a suitable and reliable agent for the cleaning of historical iron-based indoor objects presenting simple stratigraphies. When the corrosion layer is more complex, such as when it includes sediments, a preliminary mechanical cleaning or renewal of the application of DFO-amended gel may be effective.

## Conclusion

The potential of the secondary metabolites siderophores, in particular deferoxamine, was positively evaluated for iron corrosion removal in the field of heritage, with outcomes similar to the use of traditional petrochemical EDTA. It demonstrated the possibilities to go toward a practice with less health and environmental concerns and no compromise related to the efficacy of treatment. Their use in naturally originated hydrogels was possible although a preliminary fine-tuning of the application parameters may be performed according to the intended intervention. Optimal cleaning with the DFO-amended agar gel was achieved when it was applied hot, at a concentration not lower than 3×10^−2^ M and with frequent reiteration of the treatment, which also allows better control of the treatment by the operator. In addition, the use of deferoxamine at neutral pH allows a more lenient post-treatment rinsing step, in opposition to EDTA which has an acidic pH.

Consideration to the environmental and economic aspects is also crucial, taking into account the production of the DFO-agar agent to its final use (time of application, reiteration, etc.), comparatively to Na_2_EDTA.

A further look into siderophore types, their different stability constants with iron and other ions, could help sharpen the cleaning performances, by targeting eventual other compounds, such as sediments, present on the objects.

## Data Availability

The datasets generated during and/or analyzed during the current study are available from the corresponding author on reasonable request. This manuscript has associated data in a data repository. [Authors’ comment: Data repositoty is available under OLOS portal (https://access.olos.swiss/portal/home).]
